# A Compact and Fast Single‐Spike TTFS Encoder With Ultralow Operating Voltage for Efficient Neuromorphic Fourier Transform

**DOI:** 10.1002/advs.78217

**Published:** 2026-10-15

**Authors:** Yang Wu, Qi Wei, Junfeng Zhang, Lirou Su, Ning Mao, Beibei Huang, Jiaying Chen, Dian Huang, Shengzhong Feng, Yishu Zhang, Shuai Zhong

**Affiliations:** ^1^ Guangdong Institute of Intelligence Science and Technology Hengqin Zhuhai Guangdong China; ^2^ ZJU‐Hangzhou Global Scientific and Technological Innovation Center College of Integrated Circuits Zhejiang University (ZJU) Zhejiang China

**Keywords:** Fourier Transform, single spike, sub‐microsecond coding speed, TTFS encoder, ultralow operation voltage

## Abstract

Fourier transform (FT) is an indispensable mathematical foundation for frequency‐domain analysis in modern signal processing. However, conventional FT hardware relies on complex digital logic circuits, leading to high power consumption and poor adaptability. Spiking neural networks (SNNs) with time‐to‐first spike (TTFS) encoding offer advantages in energy efficiency, yet existing TTFS encoders suffer from high operating voltage, slow encoding speed, complex peripheral circuits, and redundant spike outputs. Here, we propose a compact, ultralow‐voltage, and ultra‐fast single‐spike TTFS encoder based on a Pt/TaO_x_/V device. The device exhibits reliable threshold switching, for which the combined experimental and computational results support a proposed mechanism coupling oxygen vacancies‐assisted conduction in TaO_x_ with a thermally driven conductivity change in a VO_2_‐like interfacial region. Moreover, it achieves single‐spike TTFS encoding at an ultralow voltage of ∼0.35 V with sub‐microsecond (∼100 ns) encoding speed in a simplified circuit configuration. Systematic characterizations on multi‐tone signals, digitized speech segments, and linear frequency‐modulated non‐stationary signals demonstrate that the SNN‐FFT system achieves spectral fidelity highly consistent with standard FFT, while maintaining strong sparsity and low power consumption. This work not only develops a high‐performance TTFS spike encoder but also provides a feasible and efficient solution for low‐power, real‐time, miniaturized neuromorphic FT hardware.

## Graphical abstract

A Pt/TaO_x_/V single‐spike TTFS encoder achieves ultralow‐voltage (∼0.35 V), high‐speed (∼100 ns) operation with simplified circuitry. Integrating bipolar nonuniform temporal encoding with a spiking‐DFT neuron model enables a neuromorphic FFT framework. Device‐calibrated simulations demonstrate high spectral fidelity across multitone, speech, and chirp signals, offering a promising route toward low‐power, real‐time neuromorphic FFT signal processing.

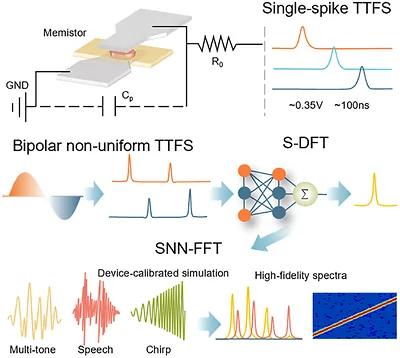

## 1 Introduction

Fourier transform (FT)serves as a foundational mathematical framework in signal processing, facilitating the conversion of signals from the time domain to the frequency domain. This transformation is essential for extracting intrinsic frequency‐domain characteristics across a wide range of disciplines, including telecommunications, medical imaging, and spectral analysis. For digital hardware systems, the discrete Fourier transform (DFT) provides a computable formulation by converting n time‐domain samples into n frequency components. However, its *O(n^2^)* computational complexity severely limits scalability for large datasets. The development of the fast Fourier transform (FFT) represented a pivotal algorithmic breakthrough, exploiting the symmetry and periodicity of complex exponentials to reduce the complexity to *O(nlogn)*, thereby enabling real‐time spectral analysis in practically all modern digital systems [1, 2]. Subsequent algorithmic refinements have been proposed to optimize computational efficiency, including split‐radix and sparse FFT variants [3, 4].

Despite these notable algorithmic advantages of the FT, its hardware implementation is plagued by inherent challenges. Traditional FT hardware relies on intricate digital circuit architectures, which are associated with high power consumption and area overhead. These constraints make conventional FT implementations incompatible with energy‐constrained scenarios, such as edge computing and wearable electronic devices. In recent years, artificial neural networks (ANNs) have been investigated as an alternative approach for FT implementation [5, 6]. Nevertheless, ANNs utilize continuous numerical signal transmission and depend on massive multiply‐accumulate operations, resulting in elevated energy consumption and limited compatibility with low‐power hardware platforms. Recently, spiking neural networks (SNNs), which mimic the information transmission mechanisms of biological neural systems, have emerged as a more viable solution for the efficient implementation of FFT [7, 8]. In comparison to ANNs, SNNs exhibit inherent event‐driven properties, which substantially reduce energy consumption and improve hardware efficiency [9, 10, 11]. A critical component of SNN‐based signal processing is the spike encoder, which converts external analog or continuous signals into spikes that can be processed by SNN neurons [12, 13]. As the interface between input signals and the SNN architecture, the encoder directly dominates the efficiency of information transmission, the accuracy of signal representation, and the overall performance of the network. Thus, spike encoding is a pivotal step of SNN design for high‐performance signal processing.

Currently, prevalent spike encoding methods for SNNs include rate coding and time‐to‐first‐spike (TTFS) coding [14, 15, 16]. Rate coding represents signal intensity by the number of spikes generated within a fixed time window; however, it requires multiple spikes to ensure encoding resolution, leading to relatively high energy consumption and prolonged computation latency. In contrast, TTFS coding encodes signal information by the time interval between the initiation of the time window and the generation of the first spike, where higher signal intensity corresponds to an earlier first spike. TTFS coding offers distinct advantages: it possesses theoretically infinite resolution, circumvents quantization errors induced by limited spike counts, reduces energy consumption by relying solely on the first spike, and enables low‐latency computation. These attributes make TTFS coding particularly suitable for high‐efficiency SNN‐based signal processing tasks, such as FT implementation.

With the advancement of hardware technologies, significant progress has been achieved in the hardware implementation of TTFS coding with transistors and emerging devices [17, 18, 19, 20, 21, 22, 23, 24, 25, 26, 27, 28, 29, 30, 31]. Among these devices, volatile memristors have garnered considerable attention due to their low power consumption, scalability, and rich dynamics, which are highly compatible with the spiking mechanism of SNNs. Nevertheless, existing volatile memristor‐based TTFS encoders still exhibit considerable room for optimization in terms of energy efficiency, area efficiency, operating voltage, and encoding speed. Most current strategies generate multiple spikes within a single time step, leading to high energy consumption and restricting their applicability in low‐power scenarios [18, 21, 26, 29]. To address this issue, recent research efforts have achieved single‐spike TTFS encoding within a single time step, which significantly reduces energy consumption by eliminating redundant spike generation [25, 28, 30]. However, these single‐spike TTFS encoders rely on complex peripheral control circuits, which increases hardware complexity and area overhead, thereby impeding their practical deployment. In addition, the operation voltage (> 1 V) and the TTFS coding speed (∼ millisecond) also need further improvement to make TTFS encoders more energy efficient and faster. Therefore, the development of a compact single‐spike TTFS encoder with low operation voltage and fast coding speed is crucial for advancing the efficient implementation of SNN‐based FFT.

In this work, we present a compact, ultralow‐voltage, and high‐speed single‐spike TTFS encoder based on a Pt/TaO_x_/V device for efficient neuromorphic FT. The device shows uniform threshold switching (TS) behavior, originating from the collaborative mechanism of oxygen vacancy filaments in TaO_x_ and a IMT in a VO_2_‐like region, which is formed at the TaO_x_/V interface, as verified by first‐principles simulations and experimental verification. By connecting it with a resistor and precisely regulating the generated Joule heating during operation, a single‐spike TTFS encoder (∼0.35 V operating voltage, ∼100 ns encoding speed) is constructed. Based on this TTFS encoder, a bipolar nonuniform TTFS mapping strategy and a spiking‐DFT (S‐DFT) neuron model are developed to establish a SNN‐FFT framework. The performance of the SNN‐FFT system is validated using multi‐tone signals, digitized speech segments, and linear frequency‐modulated (LFM) non‐stationary signals, demonstrating high spectral fidelity that is competitive with standard FFT. This work provides a high‐efficiency spike encoding solution and paves the way for low‐power, real‐time neuromorphic FT hardware.

## 2 Results and Discussion

FT serves as a fundamental and versatile mathematical methodology for signal processing, which facilitates comprehensive frequency‐domain characterization and analysis of arbitrary signals. Specifically, FT decomposes arbitrary time‐domain signals and functions into discrete individual frequency components, thereby converting complex time‐domain signals into the linear superposition of elementary sinusoidal components, as illustrated in Figure 1a. The FT of a signal f(t) is defined as follows:

(1) $$F\;\left(\omega \right)=\int_{-\infty }^{+\infty }f\left(t\right)e^{-i\omega t}\;dt$$

where *f(t)* is the original signal in the time domain, ω represents its frequency component, and *F*(ω) is the FT operation of the signal *f(t)*, transforming the original signal *f(t)* into its frequency‐domain representation. Notably, the FT has garnered widespread attention across diverse scenarios, including partial differential equations, radar signal processing, and sound localization (Figure 1b), underscoring its indispensable role in advancing related research and applications.

**FIGURE 1:**
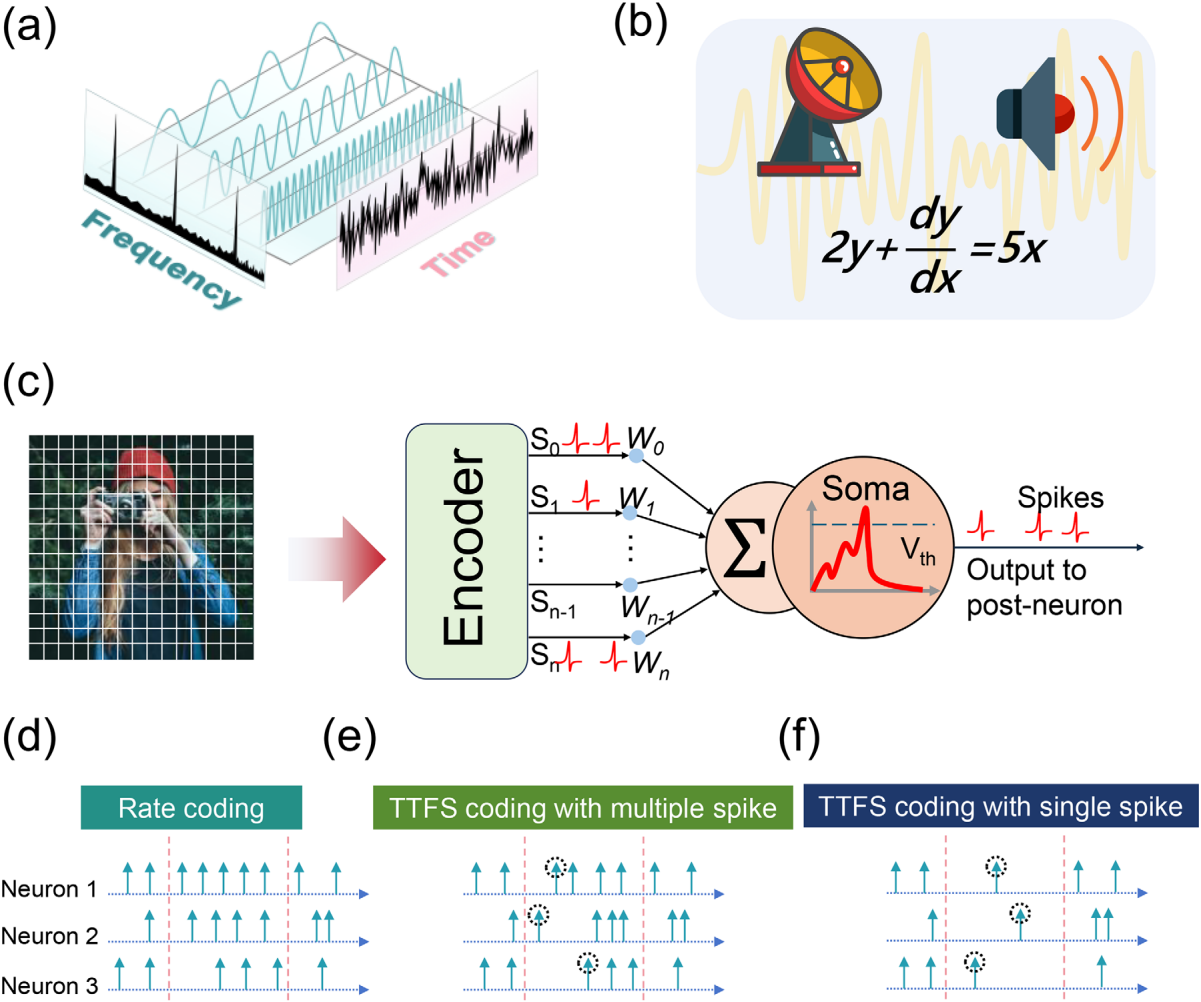
Fourier transform (FT), spiking neural network (SNN), and its encoding schemes. (a) FT is a mathematical approach to convert time‐domain signals into the frequency‐domain sinusoidal components. (b) Various applications of FT include partial differential equations, radar signal processing, and sound localization. (c) Schematic of the working flow of SNNs. The external continuous analog signals are first transformed into discrete spike trains by an encoder and subsequently fed into the neural network for decoding operations. (d) Rate coding. It encodes signal intensity by the number of spikes generated within a fixed time window. (e) TTFS coding with multiple spikes. This method generates multiple spikes within a single time step but uses the timing of the first spike to encode signal intensity, which is energy inefficient. (f) TTFS coding with a single spike. This approach generates only one spike within a single time step and uses the timing of this spike to encode signal intensity, which is sparse and energy efficient.

Although demonstrating high efficacy, the practical implementation of the FT usually involves considerable power consumption. SNNs stand out as a viable alternative, as shown in Figure 1c. In particular, SNN‐based approaches to FT possess distinct merits. SNNs can inherently perform the matrix multiplication operations, which are demanded for FT, via fully connected or sparsely connected multilayer spiking networks, thereby eliminating the necessity for complex circuitry. Moreover, neural encoding schemes transform continuous input signals into discrete spike trains, allowing SNNs to approximate FT operations using binary spike events rather than energy‐intensive floating‐point computations. In this regard, the encoding strategy is of paramount importance. Compared with rate coding and multi‐spike TTFS coding (Figure 1d,e), single‐spike TTFS coding minimizes spike activity and thus offers superior energy efficiency (Figure 1f).

The fabricated TaO_x_ device features a sandwich structure with an active hole diameter of 50 µm, and a detailed process can be found in Figure S1. A cross‐sectional transmission electron microscopy (TEM) image of the device is illustrated in Figure 2a, which confirms the stacked Pt/TaO_x_/V layered structure, and the thicknesses of the Pt, TaO_x_, and V layers were approximately 45, 10, and 60 nm, respectively. The distribution of the Pt, Ta, O, V elements was determined by using energy‐dispersive x‐ray spectroscopy (EDS) with mapping and line scanning results, as presented in Figure S2 and S3. The Ta 4f spectrum of the TaO_x_ film is shown in Figure 2b. Three peaks can be obtained by peak fitting: peaks at 26.15 and 28 eV correspond to Ta^5+^ 4f_7/2_ and Ta^5+^ 4f_5/2_, while the peak at 24.8 eV corresponds to Ta^4+/3+^. This implies there is sub‐stoichiometric tantalum oxide in the film. Figure 2c presents the O 1s XPS spectrum of the TaO_x_ sample, with two distinct fitted peaks observed at 530.4 and 531.3 eV, corresponding to lattice oxygen (O_L_) and non‐lattice oxygen (O_V_), respectively. Based on the area ratio of these two peaks, the percentage of non‐lattice oxygen in the TaO_x_ layer was calculated to be 27.12%, which indicates the presence of an amount of oxygen vacancies and structural defects within the TaO_x_ [32, 33]. Figure 2d presents the bipolar *I–V* characteristics recorded over 600 switching cycles. The device maintains threshold switching (TS) under both voltage polarities. The device transitions from a high resistance state (HRS) to a low resistance state (LRS) when the applied voltage exceeds a threshold voltage (V_th_) of ∼ 0.23 V. Conversely, the device reverts to the HRS when the applied voltage drops below the holding voltage (V_hold_) of 0.17 V. Figure 2e displays cumulative distribution plots of the positive and negative threshold/holding voltages (i.e., V_th_, V_hold_, −V_th_, and −V_hold_) across 600 repeated cycles. The coefficient of variation (C_v_), defined as the ratio of the standard deviation (σ) to the mean value (µ), was 1.3%, 0.5%, 1.3%, and 0.5% for V_th_, V_hold_, −V_th_, and −V_hold_, respectively, demonstrating low cycle‐to‐cycle (C2C) variations. The device‐to‐device DC performance of the Pt/TaO_x_/V device can be found in Figure S4. Figure 2f illustrates the endurance performance of the device over 600 cycles. The resistance states remain clearly separated, with no significant fluctuations detected.

**FIGURE 2:**
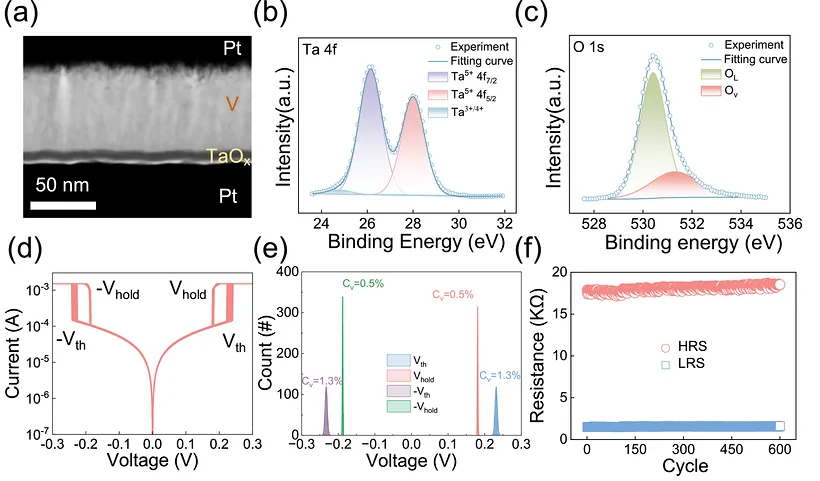
Materials characterization and electrical testing of the memristor. (a) SEM image of Pt/TaO_x_/V device. High‐resolution XPS spectra of (b) Ta 4f and (c) O 1s of TaO_x_ films. The results indicate that there are oxygen vacancies in the TaO_x_ thin film. (d) *I–V* characteristics of the Pt/TaO_x_/V device repeated for 600 cycles. (e) Cumulative distributions of positive threshold voltage (V_th_), positive holding voltage (V_hold_), negative threshold voltage (−V_th_), and negative holding voltage (−V_hold_). (f) The distribution of the Pt/TaO_x_/V device at high and low resistance states (HRS, LRS).

Vanadium oxides undergo an insulator‐to‐metal transition (IMT) at their phase transition temperature. This intrinsic property has been harnessed by researchers to fabricate TS devices, where Joule heating is employed to induce resistance modulation in these materials [34, 35]. To further examine the proposed interfacial mechanism, cross‐sectional EELS characterization was performed (Figure S5). The elemental maps resolve the oxygen‐containing oxide region and the adjacent V electrode. Position‐resolved spectra reveal that at the TaO_x_/V interface, the V‑L_2_ white line exhibits a noticeable chemical shift toward higher energy loss relative to metallic V, confirming partial oxidation of V and VO_x_ formation at the interface. Temperature‐dependent electrical measurements provide complementary evidence concerning the switching process. Increasing the ambient temperature from 298 to 328 K shifts the switching features toward lower applied voltages. These observations indicate that elevated temperature facilitates the threshold switching. The interfacial characterization and electrical measurement results support a possible IMT in a VO_2_‐like interfacial region. Based on this phenomenon, we hypothesize that VO_2_ forms within the Pt/TaO_x_/V device (at the interface of TaO_x_/V), and this VO_2_ phase is partially accountable for the observed TS behavior due to its IMT response during electrical operation. To support the formation of VO_2_ at this interface, first‐principles simulations were performed based on the λ‐Ta_2_O_5_ (001)/V (001) slab model. The charge density difference in Figure 3a reveals pronounced charge redistribution at the Ta_2_O_5_/V interface, with electrons accumulating in the interfacial region, indicating substantial charge transfer from the V layer to the adjacent oxide. V─O pair radial distribution function (RDF) before and after structural relaxation (Figure S6) shows the V─O peak shifts markedly from ∼2.62 to ∼1.91 Å, matching well with the typical peak of VO_2_. Then ab initio molecular dynamics (AIMD) simulations at 300 K were performed by depositing six V atoms onto a λ‐Ta_2_O_5_ (001) slab (denoted as V_6_@Ta_2_O_5_), mimicking the experimental sputtering deposition process. As shown in Figure 3b and Movie S1, upon contact with the Ta_2_O_5_ surface, interfacial O atoms spontaneously migrate toward the deposited V atoms, forming V─O clusters on the surface. We then compared the Bader charges of the V atoms with those in bulk V_2_O_3_, VO_2_, and V_2_O_5_ references to determine the oxidation state of these clusters. A linear fit to the three reference systems (R^2^ = 0.991) establishes a quantitative calibration between Bader charge and corresponding oxidation state (Figure 3c). Projecting the measured charge of V atoms in the V_6_@Ta_2_O_5_ model onto this calibration line yields an inferred oxidation state of approximately +4. Together with the shortened V─O distances and oxygen redistribution observed in the simulations, this result is consistent with a VO_2_‐like local chemical environment within the interfacial region.

**FIGURE 3:**
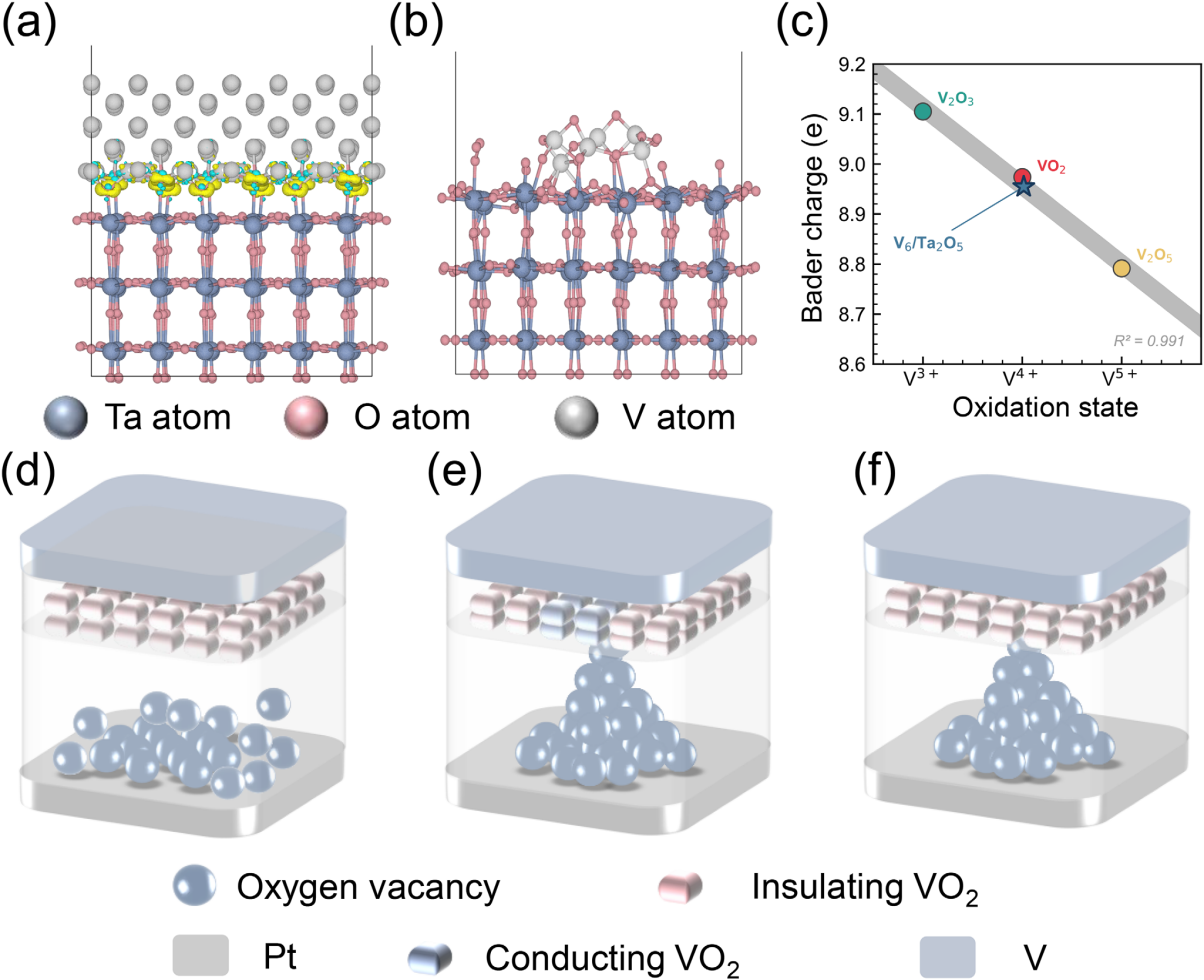
(a) Charge density difference of Ta_2_O_5_/V interface. The yellow and cyan areas indicate electron accumulation and depletion, respectively, and the value of the isosurface is 0.02 e/Å [3]. (b) Snapshots from AIMD simulation of V_6_@Ta_2_O_5_. (c) Bader charge of the vanadium oxidation state. Three bulk oxides (V_2_O_3_, VO_2_, V_2_O_5_) serve as references; the V_6_/Ta_2_O_5_ projects to +4. (d) Initial state of the Pt/TaO_x_/V device. An ultrathin VO_2_ layer is formed at the TaO_x_/V interface. (e) Under the condition of V > V_th_, a conducting pathway involving oxygen vacancies filament (TaO_x_ layer) and metallic VO_2_ (VO_2_ layer) is formed to bridge the top electrode and bottom electrode. (f) Under the condition of V < V_hold_, the metallic VO_2_ is converted to insulating VO_2_, while the oxygen vacancies filament in the TaO_x_ layer is retained.

Therefore, the resistance switching mechanism of the fabricated memristor can be elaborated as follows (Figure 3d–f): during positive voltage sweeping, oxygen ions within the TaO_x_ layer migrate toward the top electrode under the applied electric field, leading to the buildup of oxygen vacancies in the vicinity of the bottom electrode. As the voltage increases, oxygen vacancies continue to accumulate and ultimately form conductive pathways that connect the top and bottom electrodes, resulting in an abrupt increase in device current. Once these conductive filaments are established, a further increase in the applied voltage drives a large current through the filaments, generating substantial Joule heat that triggers the IMT of the VO_2_ layer, causing the TS phenomenon. As the sweeping voltage decreases, the device current reduces, and Joule heat dissipates, prompting VO_2_ to revert from the metallic state to the insulating state and causing a corresponding abrupt decrease in current. However, because the oxygen vacancy‐derived conductive filaments remain intact, the device resistance does not fully return to its initial HRS, and the current fails to immediately drop to its original LRS. Similarly, upon the application of negative bias, oxygen vacancies accumulate at the top region to form conductive pathways. As the bias voltage increases, VO_2_ undergoes an insulator‐to‐metal transition accompanied by TS behavior. When the applied voltage decreases, vanadium oxide reverts to its insulating state, leading to a sharp drop in current.

The TTFS encoder was then achieved based on this Pt/TaO_x_/V memristor. Figure 4a illustrates the configuration of the proposed TTFS encoder circuit, in which the Pt/TaO_x_/V memristor operates in its TS mode. A resistor (R_s_) with a resistance value of 1786 Ω is incorporated into the circuit in series with the memristor, and the device's intrinsic parasitic capacitance (C) is also involved into the circuit setup. During electrical testing, the input voltage is applied to the node before the memristor, while the resulting current spikes are recorded at the grounded terminal. The removal of external capacitors simplifies the circuit topology and enhances integration density, a feature that is highly beneficial for the implementation of neuromorphic hardware systems. Figure 4b displays the device's current response under an applied voltage of 0.3 V. Based on the energy estimation protocol, the energy per spike is around 0.72 nJ, and a comparison table including energy per spike, area, endurance, variability, and circuit implementation is shown in Table S1. Different from conventional oscillatory encoders, only a single current spike is produced over a stimulus duration of 10 µs, which is most likely achieved by precise control, through pulse configuration, of the Joule heat required to deliver one current spike. Noted that the current spike is abrupt and irregular, suggesting that its formation is not associated with the periodic integration and relaxation processes responsible for the operation of oscillatory encoders. For the designed circuit configuration, under a constant DC bias, the memristor initially stays in HRS and sustains almost the entire applied voltage owing to its dominant resistance, leading to an approximately static voltage division between the memristor and the series resistor. Despite the lack of an externally increasing voltage, the sustained electrical stress induces gradual local thermal accumulation within the memristor, which shifts the device's internal state toward its switching threshold. Once this critical threshold is attained at a specific instant, the memristor undergoes an abrupt transition from HRS to LRS through TS. After switching occurs, the resistor dominates the voltage drop, rapidly reducing the voltage across the memristor to below its holding voltage and facilitating its reset to HRS. This reset mechanism allows the memristor to be re‐biased at a high voltage and undergo subsequent switching events. As shown in Figure 4c, increasing the applied voltage significantly reduces the timing of the single spike. As previously discussed, spike generation is triggered by the IMT process of VO_2_, which is governed by Joule heating. The equation of Joule heating (Q), if assuming the voltage (V), resistance (R) are constant, can be described as

(2) $$Q=V^{2}\;T/R$$

where T is time. It can be readily inferred that elevating the applied voltage effectively shortens the time required to achieve the same level of Joule heating, aligning with the experimental results. Statistical analysis of single spikes under each applied voltage was also conducted, as presented in Figure 4d and Figure S7. Evidently, spike timing exhibits stochastic behavior, and this randomness can be suppressed as the applied voltage increases. As mentioned, the stochasticity of spike timing stems from the intrinsic fluctuation in the Joule heating required during the IMT process.

**FIGURE 4:**
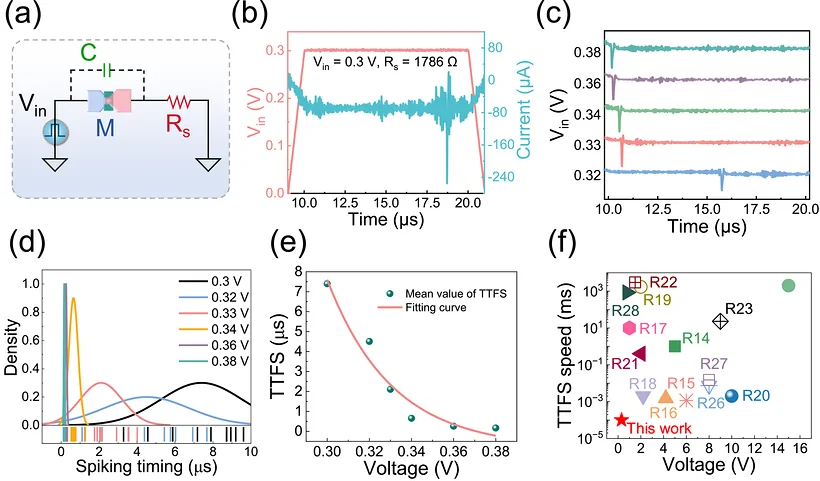
TTFS coding with a single spike based on a Pt/TaO_x_/V device. (a) Illustration of circuit design for implementation of a TTFS encoder with a single spike. (b) Current response of the device under an applied voltage of 0.3 V, and the series resistance is 1786 Ω. It can be found that only one spike is generated during the stimulus. (c) The single‐spike timing as a function of applied voltage. (d) Statistic distribution of single‐spike timing with ten tests. (e) The effect of applied voltage on the mean value of single‐spike timing. Increasing the intensity of voltage leads to a decrease in single‐spike timing. (f) Comparison of our device with previous work with respect to operation voltage and TTFS coding speed.

According to Equation (2), if the variation in Joule heating (Q) is fixed, the variation in spike timing (T) is inversely proportional to the increase in applied voltage. By extracting the mean value of TTFS, the relationship between TTFS and applied voltage can be fitted, which can be leveraged as the TTFS coding scheme for subsequent Fourier transform operations (Figure 4e). Notably, in comparison to previously reported TTFS encoders, the proposed single‐spike encoder not only features a compact structure but also exhibits ultrafast operation speed (the definition is explained in Figure S8) and an ultralow operating voltage, rendering it highly competitive (see Figure 4f and Table 1). To evaluate the reproducibility of TTFS encoding across devices, repeated latency measurements were performed on nine devices at multiple input conditions (Figures S9–S11). All devices exhibit an overall decrease in mean spike latency with increasing input voltage, demonstrating reproducible input‐dependent encoding functionality. However, variations in spike timing between trial‐to‐trial were also observed. Small operating voltages generally exhibit larger absolute timing fluctuations, whereas high voltages show smaller standard deviations. This trend may reflect enhanced latency sensitivity near the firing condition. Specifically, a slower approach to the switching threshold converts small state or threshold variations into larger timing deviations. Stronger driving accelerates the triggering process, potentially through increased local Joule heating, thereby shortening the mean latency and reducing its sensitivity to comparable perturbations. It should be noted that such variations can be alleviated by optimizing the fabrication process.

**TABLE 1:** Comprehensive comparison of emerging TTFS encoders in terms of device structure, circuit configuration, operation voltage, TTFS speed, and coding scheme. ^*^ The fast timing of TTFS and its corresponding voltage were extracted. M: Memristor, R: Resistor, C: Capacitor, T: Transistor, DRAM: Dynamic random access memory, TMS: TTFS with multiple spikes, TSS: TTFS with single spike.

Device Structure	Circuit Configuration	Operation Voltage (V) ^*^	TTFS Speed (ms) ^*^	Coding Scheme	Ref.
Pt/AlO_x/_V	1M1R	8	0.0075	TMS	[29]
Si/Al_2_O_3_/PVA/Ag	1M	2	1700	TMS	[22]
MoS_2_ transistor	1T	5	1	TMS	[17]
Pt/NbO_2_/Pt	1M1T	6	0.00116	TMS	[18]
Pt/NbO_x_/Pt	1M1T	4.2	0.0015	TMS	[19]
Pt/TaO_x_/V	1M1R	2.2	0.002	TMS	[21]
Pt/VO_x_/Pt	1M1R	10	0.002	TMS	[23]
Pt/ZnO/Ag	1M1R1C	2	0.4	TMS	[24]
Pt/TaO_x_/IGZO/Ta	1M1R+control circuit	9	23	TMS	[26]
ITO/Al_2_O_3_/ZnO:NPs/Ag	1M	0.8	850	TMS	[31]
IGZO transistor	1T	15	2000	TSS	[28]
Ti/VO_2_/Ti	1M1R1C+ control circuit	8	0.015	TSS	[30]
MOSFET	1T+control circuit	1	10	TSS	[20]
MoS_2_ transistor	DRAM+inverter	1.5	3000	TSS	[25]
Pt/TaO_x_/V	1M1R	0.3	1E‐4	TSS	This work

Figure 5 illustrates the proposed TTFS‐based encoding framework for SNN‐FFT. Figure 5a depicts the cascaded architecture and end‐to‐end signal processing workflow of the SNN‐FFT system. Input signal frames X_1_, X_2_, …, X_n_ are first projected onto the membrane potential layers of excitatory (RE) and inhibitory (IM) neurons via synaptic connections, separately encoding the positive and negative components of the raw signal. Subsequently, the inverse decoding module F(v) reconstructs frequency‐domain components in the form of (RE_i_^2^+IM_i_^2^), which achieves dual‐channel decomposition and information decoupling of the original time‐domain signal. Figure 5b characterizes the intrinsic encoding properties of conventional TTFS‐FFT, highlighting spike‐timing randomness (i.e., temporal jitter) induced by inevitable hardware device fluctuations. As the normalized input amplitude increases, the quantified timing jitter range (mean ± standard deviation) exhibits a pronounced nonlinear decline around a normalized amplitude of 0.34. This phenomenon reflects the complex nonlinear dynamics of biological‐inspired neuron models. Specifically, spike timing maintains relatively high stability under low‐amplitude input, whereas remarkable random fluctuations emerge when the input amplitude approaches intrinsic threshold boundaries. Such logarithmic nonlinear distortion of spike timing will introduce spurious harmonic components during FT, thereby degrading spectral fidelity and inducing severe spectral leakage. To address this limitation, we propose a bipolar nonuniform TTFS encoding scheme to linearize the amplitude‐to‐latency mapping and suppress device‐induced timing distortion. Figure 5c clarifies the mapping principle of real‐valued signals to the temporal axis under the proposed bipolar encoding strategy. This mechanism encompasses normalized input values spanning from −1 to 1 along the amplitude dimension. Through a dedicated time‐expanded nonlinear transformation, signal amplitudes are converted into spike delay trajectories that converge to a unified temporal reference node (e.g., t = 1.0). The gray shaded region on the right denotes an over‐threshold steady‐state protection zone to prevent abnormal spike firing. In contrast to the local nonlinear fluctuation observed in Figure 5b,d presents the linearized mapping performance enabled by bipolar nonuniform TTFS encoding. In this optimized encoding paradigm, the normalized timing jitter exhibits a strongly linear negative correlation with input amplitude. When the normalized input approaches zero (corresponding to a baseline voltage of 0.3 V), temporal jitter decreases to its minimum, explicitly defining the effective linear dynamic range of the proposed encoder. This reveals a fundamental advantage of the designed encoding mechanism: spikes fire with higher priority and narrower jitter windows under low signal voltage, while lower firing priority and wider jitter windows occur at high signal voltage. This amplitude‐dependent jitter characteristic provides a solid theoretical basis for subsequent anti‐noise interval division and nonuniform tolerance partitioning, as further detailed in Note S1 and Figure S12. Inspired by the dual firing modality of thalamocortical neurons, wherein both depolarization via positive current and hyperpolarization via negative current can elicit spike generation, we establish a spatiotemporal evolution model for the S‐DFT neuron membrane potential, as shown in Figure 5e. This model converts computationally intensive DFT operations into hardware‐friendly TTFS amplitude‐to‐temporal‐latency mapping. The membrane potential evolution is distinctly divided into two sequential stages. Silent integration stage (0 to t_max_): The neuron operates in a spatial integration mode to accumulate weighted input signals from presynaptic synapses. The orange and blue curves, corresponding to positive and negative signal components respectively, demonstrate discrete stepwise polarization of the membrane potential. Adopting a modified non‐leaky integrate‐and‐fire (IF) rule, the neuron is prohibited from generating spikes during this integration window. This design ensures that the DFT‐weighted sum carrying spatial information is fully converted into proportional increments of membrane potential amplitude. Spike firing stage (t_max_ to 2t_max_): The neuron switches to a decode‐and‐transmit mode governed by a symmetric bipolar threshold mechanism with paired trigger thresholds u_th_ and ‐u_th_, where u_th_ is defined as the maximum absolute value of the membrane potential at t_max_. A higher positive membrane potential rapidly reaches *u_th_* to evoke an excitatory spike, with larger input amplitude corresponding to shorter spike latency. Similarly, negative membrane potential evolves toward −u_th_ to generate inhibitory spikes with latency positively correlated to input amplitude magnitude. Overall, to bridge hardware‐level physical dynamics with algorithm‐level signal processing, we established an experimentally calibrated SNN‐FFT framework. Specifically, the fundamental single‐spike encoding characteristics, including voltage‐dependent spike latency and intrinsic timing jitter (Figure 5b–d) were directly extracted from empirical measurements on our fabricated Pt/TaO_x_/V devices. By embedding these measured device characteristics, the spatiotemporal integration dynamics of S‐DFT neurons and the end‐to‐end frequency analysis pipeline (Figure 5a,e) were subsequently implemented via behavioral system‐level simulations.

**FIGURE 5:**
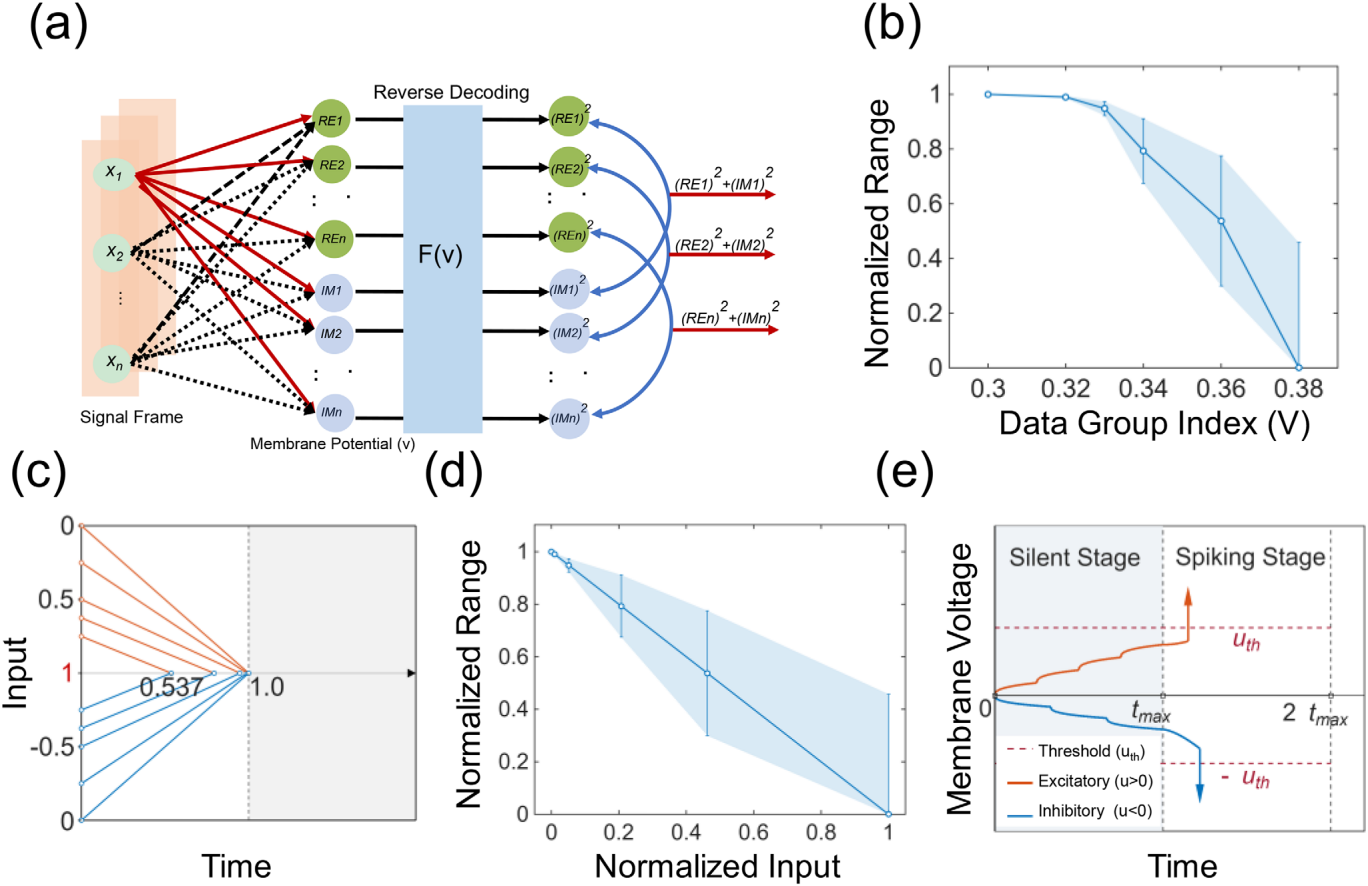
Framework and implementation breakdown of the data‐driven SNN‐FFT encoding system. (a) Cascaded architecture and signal processing pipeline of the SNN‐FFT framework. (b) Experimentally measured intrinsic spike‐timing stochasticity: timing jitter characterization induced by inherent device fluctuations. (c) Bipolar encoding via time‐extended nonlinear mapping, where positive and negative signal polarities are mapped by temporal window expansion. (d) Experimentally extracted linearized TTFS mapping characteristics: the correlation between input magnitude and spike latency within the linear dynamic range. (e) Simulated temporal evolution dynamics of the S‐DFT neuron membrane potential based on experimentally calibrated device models. Silent stage: spatiotemporal integration, in which bipolar presynaptic spikes drive stepwise membrane polarization and enable latent accumulation of input information. Spiking stage: amplitude‐to‐latency conversion, where the integrated membrane potential is modulated by bidirectional thresholds to map spatial signal magnitudes into precise output spike timings.

To quantitatively evaluate the spectral fidelity of the proposed SNN‐FFT framework, we first conduct steady‐state validation using multi‐tone synthetic signals. The encoding procedure and spectral reconstruction results for multi‐tone signals are illustrated in Figure 6a–c, which verify the high‐fidelity performance of SNN‐FFT for ideal stationary signals. Figure 6a shows the time‐domain waveform of the synthesized multi‐tone signal, which is decomposed into positive (red) and negative (blue) branches to facilitate separate polarity processing by spiking neurons. This design fundamentally resolves the inherent limitation of conventional SNNs in directly representing and processing negative signal values. Figure 6b presents the spike latency raster generated by the proposed nonuniform TTFS encoding, which serves as the core of the SNN‐FFT paradigm. Signal amplitude is uniquely encoded into the temporal order of spike firing, and the sparse spatiotemporal distribution of red and blue spike sequences demonstrates that the system achieves high numerical precision while maintaining exceptional energy efficiency. The results in Figure 6c shows that within the frequency range of 0–150 Hz, the spectrum reconstructed by SNN‐FFT (orange curve) agrees well with the standard FFT baseline (blue curve), without observable harmonic distortion or spectral leakage. We further evaluate the robustness of SNN‐FFT using digitized speech signals with inherent randomness and transient temporal characteristics. Compared with stationary multi‐tone signals, speech waveforms in Figure 6d possess more complex envelope variations and rapid phase fluctuations. Although the SNN‐FFT spectrogram exhibits slightly elevated background noise relative to the standard FFT counterpart (See Figure 6e,f) due to stochastic spike timing and quantization error, dominant spectral peaks and pitch envelopes are well preserved and highly consistent with the baseline. Finally, we investigate the two‐dimensional time‐frequency representation capability of SNN‐FFT for nonstationary radar echo signals. Taking a classic linear frequency‐modulated (LFM) signal as the testbench, the standard FFT spectrogram in Figure 6g clearly presents a typical triangular time‐frequency trajectory. The SNN‐FFT reconstructed spectrogram in Figure 6h faithfully recovers this complex time–frequency feature. Despite minor background noise introduced by spike‐domain quantization, key time‐frequency contours and characteristic components remain distinct and recognizable. Performance analysis of the SNN‐FFT method is presented in Note S2. In order to evaluate the reconstruction fidelity beyond qualitative spectral visualization, we incorporated comprehensive metrics across synthetic and real‐world signals, including Global Spectral Error (RMSE), Normalized Leakage Ratio (NLR), Peak Frequency Deviation (Δf), Spurious‐Free Dynamic Range (SFDR), and tolerance to device timing variability (detailed quantitative evaluations are provided in Note S3 and Figures S13–S15). For stationary multi‐tone signals, SNN‐FFT achieves precise frequency peak tracking (Δf = 0 Hz for SNR ≥ 10 dB) with a minimal global error floor (RMSE≈0.03). When processing complex broadband speech and non‐stationary chirp signals, the framework maintains strong feature preservation (RMSE ranging from 0.024 to 0.08) and robust target tracking (SFDR≈11 dB). Even under simulated hardware variability with up to 30% timing jitter, peak frequency estimation remains uncorrupted (Δf = 0 Hz). Altogether, these quantitative results confirm that while discrete spike quantization introduces a minor, controllable background error floor, the proposed data‐driven SNN‐FFT framework delivers robust spectral analysis capabilities highly comparable to standard digital FFT, establishing a viable paradigm for event‐driven neuromorphic signal processing.

**FIGURE 6:**
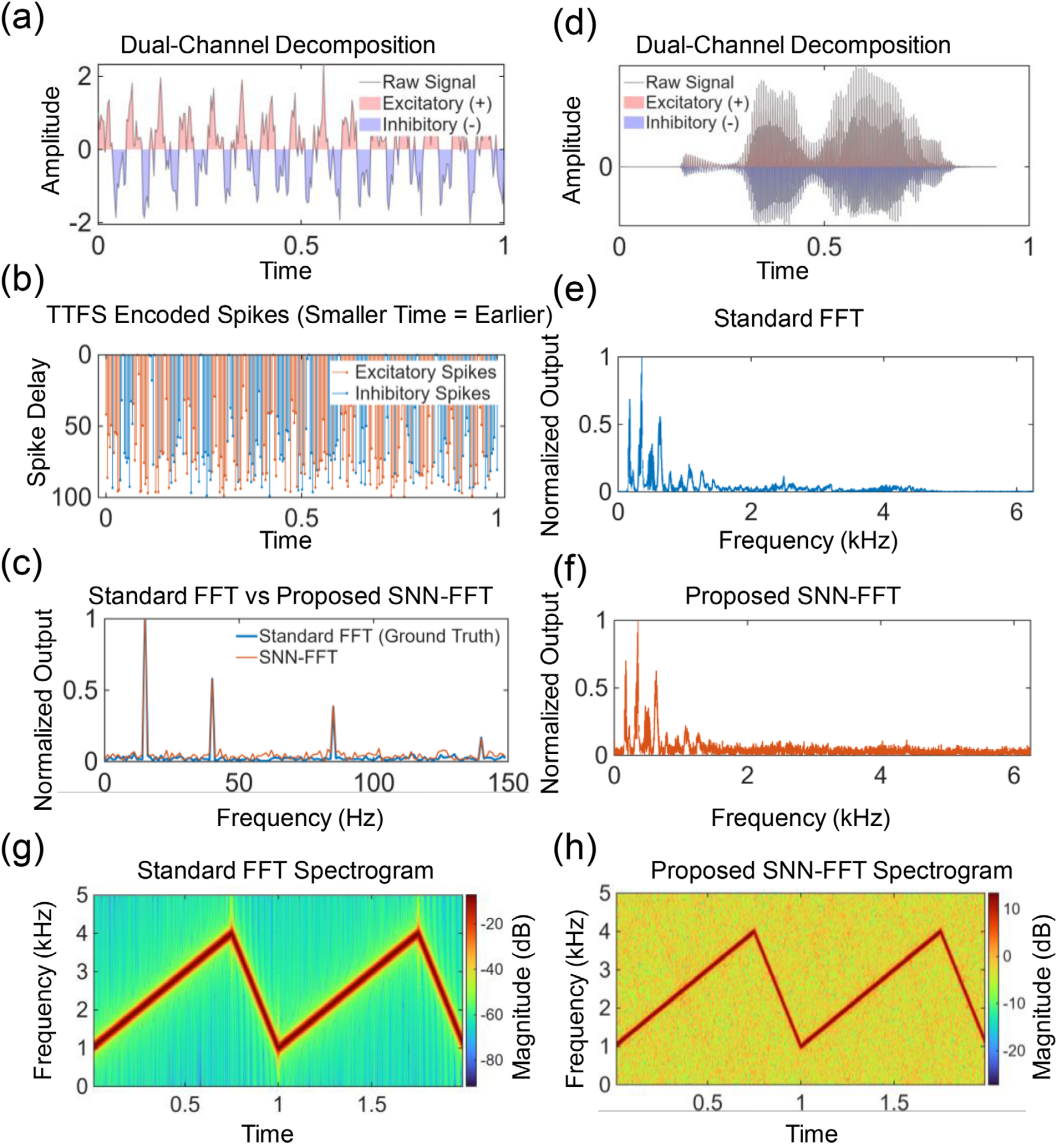
Comparative performance analysis between standard FFT and the proposed SNN‐FFT on synthetic and real‐world signals. Validation on multi‐tone synthetic signals: (a) Time‐domain waveforms and bipolar polarity decomposition; (b) Bipolar spike delay raster generated via nonuniform TTFS encoding; (c) Spectral fidelity comparison between standard FFT (baseline) and SNN‐FFT. Performance evaluation on digitized speech signals: (d) Temporal waveforms of complex speech segments; (e) Spectrogram of standard FFT; (f) Spectrogram of the proposed SNN‐FFT, demonstrating well‐preserved phonetic features despite inherent quantization noise. Time‐frequency analysis of non‐stationary signals: (g) Two‐dimensional time‐frequency representation of a linear frequency‐modulated (LFM) signal obtained by standard FFT; (h) Corresponding time‐frequency representation reconstructed by the proposed SNN‐FFT.

## 3 Conclusion

In summary, we have successfully developed a high‐performance single‐spike TTFS encoder featuring ultralow operating voltage, sub‐microsecond coding speed, and a compact circuit structure. The Pt/TaO_x_/V device exhibits reliable and uniform threshold‐switching behavior dominated by the collaborative effects of the oxygen‐vacancy filament in the TaO_x_ layer and the IMT of interfacial VO_2_. By carefully controlling the generated Joule heating with low voltage, the encoder realizes fast single‐spike encoding in a compact circuit configuration. Leveraging this advanced device, a SNN‐FFT framework with bipolar nonuniform TTFS mapping and a S‐DFT neuron model is further proposed to achieve accurate and robust neuromorphic FT. Systematic evaluations demonstrate that the SNN‐FFT system achieves spectral fidelity highly consistent with standard FFT in processing stationary multi‐tone signals, realistic speech segments, and nonstationary LFM signals, while retaining the inherent advantages of spiking neural networks, including event‐driven sparsity and ultralow power consumption. This work establishes a scalable and practical route for low‐power, real‐time, miniaturized neuromorphic signal processing systems.

## 4 Method and Experimental Section

### 4.1 Device Fabrication

First, silicon substrates were sequentially cleaned in acetone, isopropanol, and deionized water and then dried with nitrogen gas. The bottom electrode was defined, and a Ti adhesion layer (5 nm) and a Pt bottom electrode (45 nm) were deposited by magnetron sputtering. After that, a 60 nm Si_3_N_4_ insulating layer was fabricated. Finally, TaO_x_ and V layers were grown to complete the Pt/TaO_x_/V device. All patterns were finished by photolithography along with and lift‐off and magnetic sputtering.

### 4.2 Electrical Measurements and Characterization

The electrical characteristics of the memristor were measured using a Keysight B1500A Semiconductor Device Parameter Analyzer. Electrical measurements were performed in air at room temperature unless otherwise specified. The valence states of Ta and O elements in the TaO_x_ film were analyzed using a Shimadzu Axis Supra+ XPS. The microstructure of the Pt/Ta_2_O_5_/V device was characterized using a JEOL NeoARM Transmission Electron Microscope (TEM) to analyze its structural properties.

### 4.3 First‐Principles Simulation

All the density functional theory‐based first‐principles calculations are performed by using the Vienna Ab‐initio Simulation Package (VASP) with the projector augmented wave (PAW) method [36, 37, 38]. The generalized gradient approximation (GGA) formulated by Perdew, Burke, and Ernzerhof (PBE) is used as the exchange‐correlation functional [39]. The plane‐wave cutoff energy is set to 400 eV. The Monkhorst–Pack k‐point mesh with a grid spacing of 2π × 0.04 Å^−1^ is used for electronic Brillouin‐zone integration. For structure relaxation, the lattice constant and atomic positions are fully relaxed until the force on each atom is smaller than 0.02 eV/Å. For AIMD simulation, the NVT ensemble was employed using the Nosé–Hoover thermostat as implemented in VASP with a target temperature of 300 K [40, 41]. The equations of motion were integrated with a time step of 1.0 fs with the total simulation time was 1 ps. λ‐Ta_2_O_5_ was used here as it possesses a similar bandgap as the amorphous structure that has been utilized in existing devices to date. The structure was constructed according to the crystal parameters reported by Lee [42]. The Ta_2_O_5_ (001) slab and V (001) slab were used for interface construction. To ensure physical consistency and computational accuracy, the lattice mismatch between the two components was strictly controlled within 5%. A vacuum region of 15 Å was introduced along the direction perpendicular to the interface to minimize spurious interactions between the periodically repeated slabs.

### 4.4 Spike‐Based Neuromorphic SNN‐FFT Simulation

Based on experimental observations, this work develops a spike‐enabled neuromorphic FT computing framework via SNN‐FFT simulation. The proposed system adopts nonuniform bipolar spiking neurons and SNN to implement FT computation. This framework establishes a mathematically rigorous mapping between the temporal dynamics of DFT and intrinsic spiking dynamics of SNNs. Leveraging the temporal sparsity of TTFS encoding, the system achieves spectral decomposition efficiently through asynchronous spike‐driven computation, yielding a substantial reduction in dynamic power consumption. Detailed implementation methods are provided in S4.

## Author Contributions

**Yang Wu**: methodology, data curation, validation, formal analysis, visualization, writing – review and editing, writing – original draft. **Qi Wei**: methodology, software, visualization, writing – original draft, writing – review and editing, funding acquisition. **Junfeng Zhang**: software, writing – original draft, writing – review and editing, visualization. **Lirou Su**: data curation. **Ning Mao**: data curation, visualization. **Beibei Huang**: data curation. **Jiaying Chen**: writing – original draft, writing – review and editing, methodology. **Dian Huang**: writing – original draft, writing – review and editing. **Shengzhong Feng**: writing – original draft, writing – review and editing. **Yishu Zhang**: conceptualization, methodology, data curation, investigation, validation, supervision, funding acquisition, resources, writing – original draft, writing – review and editing, project administration. **Shuai Zhong**: conceptualization, methodology, data curation, investigation, validation, supervision, funding acquisition, visualization, project administration, resources, writing – original draft, writing – review and editing.

## Conflicts of Interest

The authors declare no conflicts of interest.

## Supporting information




**Supporting File 1**: advs78217‐sup‐0001‐SuppMat.docx.

**Supporting File 2**: smll76122‐sup‐0002‐Video S1.mp4.

## Data Availability

The data that support the findings of this study are available from the corresponding author upon reasonable request.
