# Supplementary material for: A Compact and Fast Single‐Spike TTFS Encoder With Ultralow Operating Voltage for Efficient Neuromorphic Fourier Transform
Source: Adv Sci (Weinh). 2026 Oct 15:e78217. Online ahead of print. doi: 10.1002/advs.78217 (PMC13670328; doi:10.1002/advs.78217)
Supplement: Supplementary file 1 [file ADVS-9999-e78217-s001.docx]

**Supporting Information**

**A Compact and Fast Single-Spike TTFS Encoder with Ultralow Operating Voltage for Efficient Neuromorphic Fourier Transform**

Yang Wu,^1,#^ Qi Wei,^1,#^ Junfeng Zhang,^1, #,*^ Lirou Su,^1^ Ning Mao,^1^ Beibei Huang,^1^ Jiaying Chen,^1^ Dian Huang,^1^ Shengzhong Feng,^1^ Yishu Zhang,^2, *^ and Shuai Zhong^1, *^

^1^Guangdong Institute of Intelligence Science and Technology, Hengqin, Zhuhai, Guangdong, 519031, China

^2^ZJU-Hangzhou Global Scientific and Technological Innovation Center, College of Integrated Circuits, Zhejiang University (ZJU), Zhejiang, 311200, China

*Corresponding authors: zhangjunfeng@gdiist.cn, zhangyishu@zju.edu.cn and zhongshuai@gdiist.cn

Figure S1. The schematic structure of Pt/TaOx/V and the detailed fabrication processes

Figure S2. Energy dispersive X-ray spectroscopy (EDS) mapping of Pt, Ta, O, V element in device.

Figure S3. Energy dispersive X-ray spectroscopy line scanning results of Pt/TaO_x_/V device

Figure S4. DC performance investigation of different devices. a) 100 DC cycles and b) cumulative distribution of V_th_pos_, V_hold_pos_, V_th_neg_, V_hold_neg_ of device 1. c) 100 DC cycles and d) cumulative distribution of V_th_pos_, V_hold_pos_, V_th_neg_, V_hold_neg_ of device 2.

Figure S5. Cross-sectional EELS characterization and temperature-dependent electrical characteristics of the Pt/TaO_x_/V device. (a) O and V elemental mapping. Scale bar: 20 nm. (b) Cross-sectional electron microscopy image indicating the three acquisition positions and the corresponding EELS spectra. The V-L_3_, V-L_2_, and O-K edges are marked. Points 1-3 progress from the TaO_x_ side toward the V electrode. (c) I-V characteristics measured at ambient temperatures ranging from 298 to 328 K.

Figure S6. V-O pair radial distribution function (RDF) before and after structural relaxation. (a) Crystal structure of Ta_2_O_5_/V before relaxation. (b) Crystal structure of Ta_2_O_5_/V after relaxation. (c) RDF of Ta_2_O_5_/V before and after relaxation, as well as VO_2_, the inset is the crystal structure of VO_2_.

Figure S7. Statistic testing of timing of spike under different applied voltages.

Figure S8. The TTFS encoding speed is defined by the timing difference between the onset of the stimulus and the spike.

Figure S9. a) Current response of device under applied voltage of 0.325 V and the series resistance is 2005 Ω. b) The single-spike timing as a function of applied voltage. Statistic testing of timing of spike under different applied voltages: c) 0.325 V, d) 0.328 V, e) 0.335 V, f) 0.345 V, g) 0.365 V. H) Statistic testing of timing of spike under different applied voltages.

Figure S10. a) Current response of device under applied voltage of 0.27 V and the series resistance is 2059 Ω. b) The single-spike timing as a function of applied voltage. Statistic testing of timing of spike under different applied voltages: c) 0.27 V, d) 0.28 V, e) 0.285 V, f) 0.29 V, g) 0.3 V. H) Statistic testing of timing of spike under different applied voltages.

Figure S11. TTFS latency statistics for nine devices. Symbols represent the mean spike latency at each tested input voltage, and error bars indicate standard deviation from repeated encoding trials. The annotated μ and σ values denote the mean latency and standard deviation, respectively.

Table S1. Comparison of previous TSS reports to this work in terms of energy per spike, area, endurance, variability, circuit implementation.

| Metric | [1] | [2] | [3] | [4] | This work |
| --- | --- | --- | --- | --- | --- |
| Energy per event | N.A | 5.27 nJ | 2.82 nJ | N.A | 0.72 nJ |
| Area or reported size | 1.5 mm^2^ | 48.4 μm^2^ | 100 μm^2^ | 0.125 μm^2^ | 2500 μm^2^ can be further improve by scaling |
| Endurance | 600 | N.A | N.A | 500 | 600 |
| variability | N.A | 0.31%/0.61% | N.A | N.A | 1.3%/0.5% |
| Circuit complexity | Dual-gate TFT +pressure sensitive film; external acquisition/control | 1 memristor + 1 resistor+1 capacitor+3 transistors, plus membrane parasitic capacitance | DRAM + inverter; capacitor refresh and timing readout | MOSFET core + gate-inhibition control; complete component count NV | 1 memristor + 1 resistor without external capacitance |

N.A: not mentioned

References:

[1] Cai et al. IGZO-Based First Spike Timing Tactile Encoders and Coupling-Enhanced Transistor Synapses for Efficient Spiking Neural Networks. Adv. Sci. 2026, 13(7), e11168.

[2] Tiw et al. An end-to-end memristive hardware system based on single-spike coding for human–machine interfaces. Nat. Electron. 2026, 9, 225–236.

[3] Wang et al. A biologically inspired artificial neuron with intrinsic plasticity based on monolayer molybdenum disulfide. Nat. Electron. 2025, 8, 680–688.

[4] Lee et al. Time-to-First-Spike Coding in Sensory Neuron for Energy-Efficient Spiking Neural Networks. IEEE Electron Device Lett. 2025, 46(8), 1421–1424.

**Note 1. Bipolar Non-Uniform TTFS Mapping and Tolerance Interval Partitioning**

Figure S12. Non-uniform tolerance interval partitioning. Discretized encoding with heterogeneous intervals for noise-robust reconstruction

To effectively mitigate the stochastic timing jitter observed in physical device characterizations, Figure S10 illustrates the discrete implementation scheme of the proposed encoding strategy, which partitions the continuous input signal range into six non-uniform tolerance intervals. This partitioning is realized via a midpoint recursive algorithm. Specifically, high-resolution (narrow) intervals are allocated to low-amplitude signal regimes (e.g., Interval 5 and Interval 6) to guarantee superior encoding fidelity, whereas progressively broader tolerance boundaries are assigned to high-amplitude regions. For instance, the width of Interval 1 is extended from 0.5956 to 1.0000. This non-uniform discretization paradigm precisely conforms to the aforementioned nonlinear and stochastic distribution profiles inherent in the hardware dynamics. Guided by the core design rule of fine-grained division for noise-vulnerable regions and coarse partitioning for dynamically stable zones, the proposed strategy ensures robust, error-tolerant signal deployment on neuromorphic hardware platforms. Furthermore, the black triangular markers highlighted in the figure represent the optimized mapping midpoints of the original input data, a configuration that maximizes the spectral feature reconstruction fidelity during subsequent neuromorphic Fourier transform operations.

Table S2: Multi-dimensional Comparison between Traditional FFT and SNN-FFT

| **Metrics** | **Traditional FFT (Von Neumann)** | **SNN-FFT (Proposed)** |
| --- | --- | --- |
| Computing Core | Floating-point MAC | Synaptic Temporal Integration & Accumulation |
| Algorithmic Complexity | $O\left( N^{2} \right) \mathrm{or} O(NlogN)$, increases with $N$ | $O(N^{2})$ (Synaptic connectivity density) |
| Computing Latency | Grows linearly/logarithmically with $N$ | $O(1)$ (Parallel temporal evolution) |
| Theoretical Precision | Extremely High (64-bit float) | Moderate (Limited by $T_{\mathrm{window}}$ quantization) |
| Robustness | Weak (Sensitive to quantization noise) | Strong (Tolerant to temporal jitter) |
| Hardware Area | Large (Resource-intensive multipliers) | Small (Multiplication-free accumulation) |

**Note 2. Performance Discussion of the SNN-FFT Method**

As illustrated in Table S2, conventional FFT architectures rely heavily on Multiply-Accumulate (MAC) units within the Von Neumann framework, where power consumption rises sharply with signal dynamic range and bit depth. In contrast, the proposed SNN-FFT adopts the time-as-computation paradigm, leveraging the temporal attributes of spikes to transform expensive continuous multiplications into low-power asynchronous synaptic accumulations. Regarding complexity, a distinction must be made between simulation environments and hardware execution. While SNN-FFT exhibits an $O(N^{2})$ connectivity complexity in CPU simulations, it demonstrates a unique $O(1)$ temporal evolution characteristic under the parallel architecture of neuromorphic hardware. Unlike traditional FFT, which must complete all butterfly stages before outputting results, the frequency response of SNN-FFT is triggered asynchronously as spikes arrive within the simulation window $T_{window}$. This asynchronous mechanism ensures a constant temporal latency, significantly accelerating response speeds in real-time applications such as radar signal processing. Although the absolute precision of SNN-FFT is constrained by the resolution of TTFS encoding and inherent temporal jitter, its robustness is of high practical value. Traditional algorithms are prone to spectral aliasing under low-bit quantization, whereas SNN-FFT mimics the Stochastic Resonance observed in biological systems by introducing random jitter, thereby enhancing the system's defense against non-stationary noise. Furthermore, by dynamically adjusting the length of $T_{window}$, a flexible, application-specific trade-off can be seamlessly achieved between computational precision and processing latency.

Figure S13. Performance evaluation of SNN-FFT and standard FFT under varying noise and device jitter for multi-tone signals. a) Global spectral RMSE under varying SNR. b) Normalized leakage ratio under varying SNR. c) Mean peak frequency error under varying SNR. d) Global spectral RMSE under device time jitter. e) Normalized leakage ratio under device time jitter. f) Mean peak frequency error under device time jitter.

Figure S13a illustrates RMSE performance across SNRs ($-10dB$ to $30dB$). Global spectral errors for both standard FFT and SNN-FFT decrease as SNR increases. In high-SNR regimes ($\geq20dB$), standard FFT error approaches zero, whereas SNN-FFT reaches an error floor of $\approx0.03$. This plateau stems from discrete spike emission timing and $\mu$-law quantization, which introduce baseline quantization noise relative to continuous-precision baselines. Figure S13b evaluates normalized leakage ratio (NLR) under noise interference. At severe noise levels ($-10dB$), environmental noise dominates the spectrum, producing a high leakage ratio ($\approx0.9$) in both methods. As SNR improves, standard FFT leakage decays rapidly to baseline. SNN-FFT leakage follows a matching decay profile before stabilizing at $\approx0.10$ above $10dB$ SNR. Figure S13c details core peak localization error ($\Delta f$). At severe SNRs ($\leq0dB$), background noise obscuration causes frequency estimation deviations in both frameworks. However, for $SNR\geq10dB$, mean frequency errors for both architectures converge to $0Hz$, confirming that global timing periodicity preserves frequency location despite amplitude quantization errors. Fixing environmental SNR at $20dB$, normal temporal jitter with variance $\sigma_{\mathrm{var}}$ ($0\%$ to $40\%$) was applied to spike emission times ($t_{s}$) to simulate hardware variations. As shown in Figure S13d, SNN-FFT RMSE increases linearly from $0.03$ to $0.05$ with increasing jitter, deviating from the ideal FFT baseline. Figure S13e illustrates background energy spreading under temporal jitter. As $\sigma_{var}$ increases, NLR displays linearly growth, reaching $\approx0.22$ at $40\%$ variance. Random perturbations on discrete spike triggers act as phase noise, widening spectral peaks and spilling energy into background bands. Figure S13f evaluates feature tracking resilience under hardware timing non-idealities. Despite amplitude degradation and energy spreading (Figures S13d, e), SNN-FFT peak frequency error remains at $0Hz$ (zero variance) up to $\sigma_{\mathrm{var}}\leq30\%$. Variance band diffusion occurs only under extreme jitter ($>30\%$). This demonstrates that SNN-FFT, constructed via statistical trigger timing in bipolar non-uniform TTFS encoding, provides robust immunity to temporal jitter.

Figure S14. Performance evaluation of SNN-FFT and standard FFT under varying noise and device jitter for broadband speech signals. a) Global spectral RMSE under varying SNR. b) Normalized leakage ratio under varying SNR. c) Global spectral RMSE under device time jitter. d) Normalized leakage ratio under device time jitter.

Figure S14a shows global spectral RMSE for broadband speech across SNRs from $-10dB$ to $30dB$. Both standard FFT (blue) and SNN-FFT (red) show substantial error reduction with increasing SNR. Due to the high dynamic range of speech, bipolar non-uniform TTFS encoding preserves low-amplitude details, enabling SNN-FFT to achieve a low error floor ($\approx0.024$) at high SNRs while matching FFT performance in low-SNR regimes ($-10dB$ to $0dB$). Figure S14b evaluates energy residue in background speech bands (amplitude $<0.05$, e.g., high-frequency attenuation and unvoiced pauses). Standard FFT leakage decays to near zero at high SNRs, whereas SNN-FFT leakage plateaus between $0.15$ and $0.20$ for $SNR>15dB$. Continuous speech harmonics combined with temporal quantization introduce a broadband background floor, causing minor energy spillover into designated background regions. Maintaining SNR at $20dB$, normal timing jitter ($\sigma_{\mathrm{var}}$ from $0\%$ to $40\%$) was introduced to evaluate hardware variance tolerance (Figure S14c). SNN-FFT RMSE rises gradually from $\approx0.025$ to $0.08$ relative to the jitter-free baseline. The narrow standard deviation band confirms that the dual-channel TTFS scheme maintains macroscopic statistical stability when processing dynamic speech waveforms under timing noise. Figure S14d illustrates energy leakage ratio under temporal jitter. Because speech reconstruction relies on phase coherence across temporal windows, random jitter acts as non-linear phase noise, broadening spectral energy and raising the background leakage ratio from $0.16$ to $0.52$. However, as indicated by the modest RMSE in Figure S14c, this leakage represents a uniform elevation of the broadband background noise floor rather than disruption of primary formant structures.

Figure S15. Performance evaluation of SNN-FFT and standard FFT under varying noise and device jitter for broadband chirp signals. a) Global spectral RMSE under varying SNR. b) Spurious-Free Dynamic Range (SFDR) under varying SNR. c) Global spectral RMSE under device time jitter. d) SFDR under device time jitter.

Figure S15a presents the global spectral RMSE for frequency-sweeping chirp signals across an SNR range of $-10dB$ to $30dB$. While both methods exhibit decreasing RMSE as the SNR increases, their behaviors diverge at high SNR levels ($>20dB$). Specifically, the standard FFT error approaches zero, whereas the SNN-FFT converges to an error floor of $\approx0.08$. Figure S15b evaluates Spurious-Free Dynamic Range (SFDR) across noise levels. At low SNRs ($<0dB$), both frameworks perform comparably. With increasing SNR, standard FFT SFDR scales linearly toward $15.5dB$, whereas SNN-FFT SFDR plateaus at $\approx11dB$ beyond $20dB$ SNR. Non-linear threshold mapping in dual-channel spike encoding excites minor harmonic intermodulation components, establishing an upper bound on dynamic range for continuous sweep signals. At a fixed $20dB$ SNR, Gaussian temporal jitter ($\sigma_{\mathrm{var}}$ from $0\%$ to $30\%$) was introduced to evaluate hardware variability effects (Figure S15c). SNN-FFT RMSE displays a modest linear increase from $0.088$ to $0.119$ compared to the jitter-free baseline. The narrow variance band confirms architectural tolerance to temporal fluctuations. Figure S15d evaluates SFDR sensitivity to hardware timing perturbations. Random jitter degrades phase alignment among spikes, elevating the background noise floor. Across jitter levels up to $40\%$, SNN-FFT SFDR decreases only slightly (from $10.1dB$ to $8.6dB$). This demonstrates that while dynamic range is bounded by encoding harmonics, primary spectral feature extraction remains resilient to temporal timing variations.

**Note 3. Quantitative Spectral Evaluation of SNN-FFT**

Standardized Evaluation across Multi-Tone, Broadband Speech, and Chirp Signal Datasets

Let $M_{c}[k]$ denote the normalized reference spectrum of the pure ground-truth signal, and $M[k]$ represent the evaluated spectrum under environmental noise or device jitter conditions, where $k\in\{1,2,\ldots,K\}$ indexes discrete frequency bins within the single-sided band. All solid lines depict mean values *(*$\mu$*)* across 100 independent Monte Carlo trials, with shaded bands illustrating standard deviation *(*$\sigma$*)* intervals.

3.1 Global Spectral Root Mean Square Error (RMSE)

To measure the overall amplitude deviation between the reconstructed spectrum and the ground-truth reference across all frequency bins:

$RMSE=\sqrt{\frac{1}{K}\underset{k=1}{\overset{K}{\sum}}{\mid M[k]-M_{c}[k]\mid}^{2}}$	(1)

where $K$ denotes the total number of discrete frequency bins within the single-sided band.

3.2 Normalized Leakage Ratio (NLR)

To evaluate the parasitic energy leakage into background spectral regions where the baseline signal amplitude is negligible, let $\Omega_{leak}$ represent the set of background frequency indices:

$NLR=\frac{\underset{k\in\Omega_{leak}}{\sum}\left( M[k] \right)^{2}}{\underset{k=1}{\overset{K}{\sum}}\left( M[k] \right)^{2}+\epsilon}$	(2)

where $\epsilon={10}^{-12}$ is a small regularization constant introduced to prevent numerical division instability.

3.3 Mean Peak Frequency Error ($\Delta f$)

To quantify the frequency tracking deviation of primary spectral peaks:

$\Delta f=\frac{1}{N_{c}}\underset{c=1}{\overset{N_{c}}{\sum}}\mid\hat{f}_{c}-f_{c}\mid$	(3)

where $N_{c}$ denotes the total number of target tone components, $f_{c}$ represents the ground-truth center frequency of component $c$, and $\hat{f}_{c}$ is the estimated peak frequency identified within the local search window $W_{c}$ surrounding component $c$.

3.4 Device Variability and Temporal Jitter Modeling

To model non-ideal hardware characteristics, such as process variations and thermal noise inherent in neuromorphic architectures, a multiplicative temporal perturbation is introduced into the spike firing timestamps. At a fixed environmental baseline of $SNR$, the jittered firing timestamp $t_{s}^{'}$ associated with an ideal emission time $t_{s}$ is formulated as:

$t_{s}^{'}=t_{s}\left( 1+\Delta t \right),where\Delta t\sim N\left( 0,\sigma_{var}^{2} \right)$	(4)

where $\Delta t$ represents a zero-mean Gaussian random variable, and $\sigma_{var}$ characterizes the relative variance of the temporal jitter, scaled from $0\%$ to $50\%$ across the experimental evaluations.

3.5 Spurious-Free Dynamic Range (SFDR)

To quantify the dynamic range ratio between the primary signal response and the maximum spurious background component for frequency-sweeping signals:

$SFDR(dB)=20{log}_{10}\left( \frac{{max}_{k\notin\Omega_{leak}}M[k]}{{max}_{k\in\Omega_{leak}}M[k]+\epsilon} \right)$	(5)

where $k\notin\Omega_{leak}$ indexes the frequency bins corresponding to the primary signal envelope, $k\in\Omega_{\mathrm{leak}}$ designates the set of non-signal baseline bins, and $\epsilon={10}^{-12}$ is a numerical regularization constant introduced to avoid singularity in division.

**Note 4.** **Implementation of the SNN-FFT** **Method**

The proposed framework establishes a mathematically rigorous mapping between the DFT and the temporal dynamics of a SNN. The specific method is as follows:

4.1. Bio-Inspired Dual-Channel Signal Rectification

Conventional DFT algorithms operate on complex or bipolar real-valued signals. However, biological neurons communicate via unipolar action potentials. To reconcile this, we employ a dual-channel decomposition inspired by the parallel ON/OFF pathways in the mammalian visual and auditory systems.

Specifically, the normalized input sequence $x[n]$ undergoes half-wave rectification to extract excitatory and inhibitory components:

$x^{+}\left[ n \right]=\max\left( 0,x\left[ n \right] \right),$	$x^{-}[n]=max(0,-x[n])$	(6)

This transformation ensures that the signal remains non-negative, preserving the biological constraint of firing rates, while enabling the full reconstruction of the original signal dynamics via $x[n]=x^{+}[n]-x^{-}[n]$. This step is critical for maintaining linearity in the subsequent synaptic integration stage.

4.2. TTFS Encoding with Stochastic Temporal Jitter

The core of the temporal transduction process lies in the TTFS encoding scheme, which maps signal amplitudes to precise spike latencies. This mimics the intensity-to-latency conversion observed in primary sensory neurons.

Linear Intensity-to-Latency Mapping. We define a normalized temporal factor $y_{p,m}[n]$, where higher signal amplitudes result in smaller $y$ values, representing prioritized (earlier) firing:

$y_{p,m}[n]=1-x^{+,-}[n]$	(7)

Stochasticity and Quantization: Real-world biological systems are inherently noisy. To improve the robustness of the model and simulate the probabilistic nature of synaptic vesicle release, we introduce a stochastic jitter $\epsilon$. The quantized temporal representation $\hat{y}[n]$ is governed by:

$\hat{y}[n]=E(y[n])=\mu_{idx}+\sigma_{idx}\cdot rand(-1,1)$	(8)

Here, the standard deviation $\sigma_{idx}$ is adaptively scaled based on the mean $\mu_{idx}$, ensuring that high-amplitude signals (low latency) maintain higher temporal precision. This is validated by empirical data, with mean values $\mu_{idx}=[0,0.5372,0.7930,0.9481,0.9901,1.0000]$, the corresponding standard deviations are $\sigma_{idx}=[0.2288,0.2379,0.1183,0.0255,0.0042,0.0025]$, demonstrating a consistent decline in temporal jitter as the mean approaches its maximum.

Temporal Scaling. The physical spike time $t_{in}$ within a simulation window $T_{window}$ is then:

$t_{in}^{+,-}[n]=T_{window}\cdot\hat{y}_{p,m}[n]$	(9)

4.3. Fourier-Orthogonal Synaptic Connectivity

The synaptic weights $W$ serve as the memory of the transform, encoding the orthogonal basis functions of the Fourier domain. Each frequency-selective neuron $k$ is connected to the input population via complex-valued synapses:

$W[k,n]=\frac{1}{\sqrt{N}}e^{-j\frac{2\pi kn}{N}}=W_{real}[k,n]+j\cdot W_{imag}[k,n]$	(10)

where $W_{real}$ and $W_{imag}$ represent the cosine and sine components, respectively. These weights facilitate the projection of the temporal spike patterns onto the frequency manifold.

4.4. Membrane Potential Dynamics in the Silent Stage

The integration phase occurs during the TTFS Silent Stage, where postsynaptic neurons act as temporal integrators. We define the effective stimulus duration $\tau[n]$ as the period during which the synapse remains active following a spike: $\tau[n]=T_{window}-t_{in}[n]$. The membrane potential $V_{k}$ of the $k^{th}$ neuron accumulates these inputs linearly. The total potential is the sum of contributions from both excitatory and inhibitory channels, weighted by the Fourier coefficients:

$V_{k}=\sum_{n=0}^{N-1}\left( W[k,n]\cdot\tau^{+}[n]-W[k,n]\cdot\tau^{-}[n] \right)$	(11)

Mathematically, this integration decomposes into real and imaginary components:

$\left\{ \begin{matrix} V_{real}[k]=\sum_{n=0}^{N-1}W_{real}[k,n](\tau^{+}[n]-\tau^{-}[n]) \\ \\ V_{imag}[k]=\sum_{n=0}^{N-1}W_{imag}[k,n](\tau^{+}[n]-\tau^{-}[n]) \end{matrix} \right.$	(12)

Crucially, because $\tau\approx T_{window}\cdot x$ under ideal encoding, the membrane potential accumulation is a direct temporal analogue of the DFT sum, effectively performing the multiply-accumulate operations of the Fourier transform in the time domain.

4.5. Output Spiking and Magnitude Reconstruction

The final stage involves the conversion of the integrated potentials back into an observable spectral representation.

In the Spiking Stage, the neurons generate output pulses once the observation window closes. The output latency $t_{out}[k]$ is determined by a linear charging model with threshold $V_{thresh}$ and membrane leakage rate $g$:

$t_{out}[k]=\frac{V_{thresh}-V[k]}{g}$	(13)

This ensures that neurons representing dominant frequencies fire earlier, enabling a time-to-frequency decoding.

The spectral magnitude $M[k]$ is reconstructed by combining the states of the real and imaginary neuronal populations. This synthesis represents the energy density at each frequency bin:

$M[k]=\sqrt{V_{real}[k]^{2}+V_{imag}[k]^{2}}$	(14)

The system provides a high-fidelity spectral analysis that is both computationally efficient and biologically plausible.
